# Factors Associated with Preterm, Early Preterm and Late Preterm Birth in Malawi

**DOI:** 10.1371/journal.pone.0090128

**Published:** 2014-03-03

**Authors:** Nynke R. van den Broek, Rachel Jean-Baptiste, James P. Neilson

**Affiliations:** 1 Centre for Maternal and Newborn Health, Liverpool School of Tropical Medicine, Pembroke Place, Liverpool, United Kingdom; 2 Department of Women's & Children's Health, Institute of Translational Medicine, University of Liverpool, United Kingdom; Baylor College of Medicine, United States of America

## Abstract

**Background:**

Assessment of risk factors for preterm birth in a population with high incidence of preterm birth and HIV infection.

**Methods:**

Secondary analysis of data for 2,149 women included in a community based randomized placebo controlled trial for the prevention of preterm birth (APPLe trial (ISRCTN84023116) with gestational age at birth determined through ultrasound measurement in early pregnancy. Multivariate Logistic Regression analyses to obtain models for three outcome variables: all preterm, early preterm, and late preterm birth.

**Findings:**

No statistical differences were noted for the prevalence of HIV infection (p = 0.30) or syphilis (p = 0.12) between women who delivered preterm versus term. BMI (Adjusted OR 0.91 (0.85–0.97); p = 0.005) and weight gain (Adjusted OR 0.89 (0.82–0.97); p = 0.006) had an independent, protective effect. Previous preterm birth doubled the odds of preterm birth (Adjusted OR 2.13 (1.198–3.80); p = 0.01). Persistent malaria (despite malaria prophylaxis) increased the risk of late preterm birth (Adjusted OR 1.99 (1.05–3.79); p = 0.04). Age <20 (Adjusted OR 1.73 (1.03–2.90); p = 0.04) and anemia (Adjusted OR 1.95 (1.08–3.52); p = 0.03) were associated with early preterm birth (<34 weeks).

**Conclusions:**

Despite claims that HIV infection is an important cause of preterm birth in Africa, we found no evidence of an association in this population (unexposed to anti-retroviral treatment). Persistent malaria was associated with late preterm birth. Maternal undernourishment and anemia were independently associated with early preterm birth. The study did not assess whether the link was direct or whether a common precursor such as chronic infection was responsible for both maternal effects and early labour.

## Introduction

Mortality in the first four weeks of life now accounts for 24–56% of all deaths among children under five and 75% of these occur in the first week of life. Of the estimated 4 million neonatal deaths each year, 99% occur in low income countries [1], and approximately 35% are attributed to preterm birth [2], making prematurity the leading direct cause of neonatal mortality. Babies born prematurely, but who survive the immediate postnatal period, have an increased risk of death and morbidity during childhood as well as delay in both growth and development compared to babies born at term [3].

Globally, around 10–11% of all births, or an estimated 15 million births per year, are estimated to be preterm [4],[5]. The incidence of preterm birth (before 37 completed weeks or 259 days of pregnancy) is around 10.6% in North America and 6.2% in Europe [4]. There are few reliable estimates from developing countries because of uncertainty around assessment of gestational age and consequent reliance on low birth weight as a proxy measure. We have previously reported rates of between 17% and 24% in rural, community based studies from Malawi among women with anemia[6] and in unselected populations of pregnant women respectively [7],[8]. Critically, these studies used ultrasound for accurate assessment of gestational age. These rates, we believe, are the highest reported in any unselected population using ultrasound. A review of reported preterm birth rates from 184 countries found Malawi to have highest rate at 18% [5].

Although the reported rates of preterm birth are highest in sub-Saharan Africa and the highest absolute number of preterm births occurs in Asia, there are very limited data on factors associated with preterm birth in these populations. Preterm birth in these settings is presumed to be strongly associated with infective morbidity because burdens of infection are high in these countries, and because there is strong evidence from *in vitro* and *in vivo* experiments to link infection with preterm labour [9],[10]. HIV infection has been assumed to be a ‘potent cause of preterm birth’ in Africa [11]. An understanding of the causes of the very high rates of preterm birth in Malawi may not only be of potential value to clinicians and policy makers in the local population, but may provide more generalisable insights to the clinical and research communities internationally.

Given the burden of co-morbidities during pregnancy for many women living in resource poor settings, it is plausible that factors associated with preterm birth will differ from those in more affluent populations with better access to health care. The development of innovative solutions for prevention rely on a better understanding of cause [12], including the importance of infection – which is recognized to be more strongly associated with earlier than later preterm birth [13].

## Materials and Methods

A secondary analysis was performed using data collected as part of the APPLe trial (trial number ISRCTN84023116).

For the APPLe trial, pregnant women attending three rural and one peri-urban health centres in Southern Malawi were randomised to a placebo-controlled trial of oral azithromycin (1 g) given at 16–24 weeks and 28–32 weeks gestation. Gestational age was determined by ultrasound before week 24. Women and their infants were followed up until 6 weeks post delivery. The primary outcome was incidence of preterm delivery, defined as <37 weeks. Secondary outcomes were mean gestational age at delivery, perinatal mortality, birthweight, maternal malaria, and anemia. Analysis was by intention to treat [8]. Since prophylactic treatment with azithromycin had no statistically significant impact on any of the outcome measures, including preterm birth and malarial status, the participants' data was pooled for secondary analysis regardless of allocated treatment group.

Pregnant women <24 weeks gestation (ultrasound dated) were recruited at their first antenatal visit (booking visit) at which time they were screened for anemia (Hb<11.0 g/dl by battery operated HemoCue device), malaria (peripheral parasitaemia on thick blood film) and syphilis (VDRL). All women who tested positive for syphilis were treated with benzyl penicillin (1 g i.m.). At the time the trial was conducted HIV testing was not mandatory and counseling and testing for HIV was available to all women who wished to be tested. Treatment to prevent maternal to child transmission was available at time of delivery as indicated.

All women received iron tablets daily (60 mg elemental iron as ferrous sulphate) with 0.25 mg folic acid and antimalarial prophylaxis (two doses of Fansidar: 500 mg sulphadoxine with 25 mg pyrimethamine). Women were seen at 4 weekly intervals until 32 weeks then 2 weekly until term. At 28–32 weeks, all women were reassessed for malaria and anemia and treated as needed. Women returned to the clinic for postnatal visits at 1 and 6 weeks. Community-based follow up was conducted for all women who failed to return to the antenatal or postnatal clinic as planned, or for women who withdrew from the study.

For this secondary analysis, three groups of women were defined: those whose pregnancy resulted in an early or late preterm birth (gestation 24–36 weeks), and those who delivered at term (37–41 weeks). Women who delivered after 41 weeks (postterm) were not included in the analysis. Preterm birth was subdivided into early preterm (24–33 weeks) and late preterm birth (34–36 weeks). All women who delivered preterm started labour spontaneously. Data on the general demographics of the mother (age, parity, BMI and gestational age at booking, weight gain between booking and 28–32 week visit), outcome of previous pregnancy and information about the index delivery, including type of delivery, place and supervision of delivery was analyzed for each group. Women found to be anemic (Hb<11.0) or severely anemic (Hb<8.0) both at booking and during the second visit, were considered ‘persistently anemic’ or ‘persistently severely anemic’. Blood tests for malaria (peripheral parasitaemia on thick blood film) were done both at the booking and second visit with women positive at both visits considered to have ‘persistent malaria’.

HIV testing was performed retrospectively (2009) on stored blood samples (including booking blood sample and sample taken at 28–32 weeks gestation) using the Biorad GENSCREEN Ultra HIV Ag-AB kit for detection of HIV p24 antigen and antibodies to HIV1 and HIV 2.

Data was analyzed using SPSS version 19. Frequencies, means and medians were used as appropriate to describe characteristics of all study participants. Women who gave birth to twins were excluded. Pearson's Chi Square was used to test for significant differences in dichotomous variables between women who delivered preterm versus term, while the Student's t-test was used to test differences for normally distributed continuous variables. The Mann Whitney U/Wilcoxon rank sum test was used to evaluate statistically significant differences among medians of variables with non-parametric distributions. This descriptive analysis was repeated for early and late preterm versus term birth. Multivariate Logistic Regression (MLR) analyses were conducted to obtain models for three outcome variables: all preterm, early preterm, and late preterm births.

For multivariate analysis, all variables for which p<0.10 in the univariate analyses of the specific outcome variable were considered important and included in the starting model for the corresponding multivariate analyses. Using the backwards model selection method, each model was constructed to initially contain such variables and any that were clinically relevant. Goodness of Fit tests (Hosmer and Lemeshow) and deviance statistics were used to select the best multivariate model for each outcome. Odds Ratios and 95% Confidence Intervals (CI) were calculated for each variable that remained in the model. The level of statistical significance for all analyses was set at α = 0.05.

Ethical approval was obtained from the College of Medicine Research Ethics Committee in Malawi. Informed signed consent was obtained from each participant after receiving a detailed explanation in the local language of the purpose of the study. In line with the Ethics Committee's instructions, HIV testing was performed retrospectively on stored blood samples only after the completion of the trial.

## Results

### Characteristics of study participants

**Figure 1:**
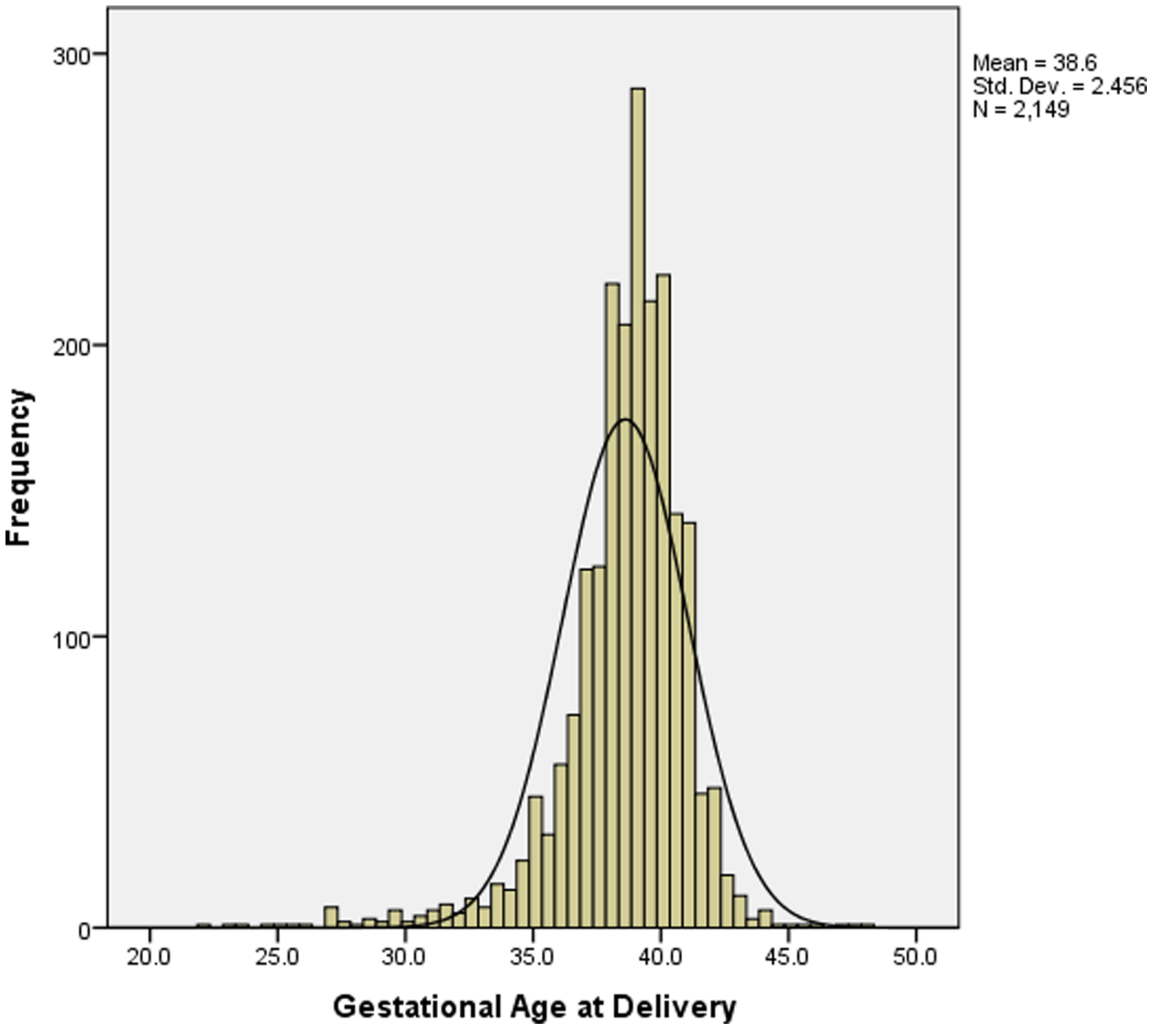
Histogram of ultrasound determined gestational age at delivery for women in southern Malawi. (n = 2,149).

A total of 2,297 women were recruited into the APPLe trial. Twenty-nine mothers who had twins were excluded from these analyses. For singleton pregnancies, data on gestation at birth was available for a total of 2,149 women. Of these 80.5% (1729/2149) delivered at term (37–41 weeks), 16.3% (351/2149) delivered preterm (<37 weeks), and 3.2% (69/2149) delivered postterm (>41 weeks gestation). Among women who delivered preterm, 75.2% (264/351) were late preterm births (34–36 weeks). Mean (SD) gestational age at birth was 38.6 (2.5) weeks. (Figure 1)

General characteristics of the study population are given in Table 1. Mean (SD) age of women included in the study was 23.0 (5.2) years and median (minimum, maximum) reported parity 1 (0,12). The majority of women in this rural population deliver in a hospital or health centre. The vast majority (93.7%) of deliveries were spontaneous vaginal deliveries, with very low rates of caesarean section (5.1%) or instrumental deliveries (forceps or ventouse) (0.4%). Breech delivery (1.9% vs. 0.6%, p = 0.02) and delivery at home (15.8% vs. 11.8% p<0.03) were both more likely to occur in women delivering preterm compared to women delivering at term.

**Table 1:** Characteristics of study population (n = 2149 women).

Study Characteristic	Characteristic Subcategory	n	%
**Age (years)**	<20	617	28.7
	0–24	861	40.1
	25–29	374	17.4
	30–34	200	9.3
	35–39	69	3.2
	40+	20	0.9
**Parity**	Primiparous (parity 0)	780	36.3
	Multiparous (parity 1–4)	1229	57.2
	Grand Multiparous (parity 5+)	140	6.5
**BMI**	BMI (mean, SD)	22.7 (SD = 2.6)	
	BMI <18.5	69	3.2
	Weight Gain (kg, n = 1502) (mean, SD)	3.3 (SD = 2.1)	
	Weight Loss (kg, n = 152) (mean, SD)	2.0 (SD = 1.4)	
**Prevalence of Morbidity**	Anemia (Hb<11.0)	1324	61.6
	Persistent Anemia	436	21.4
	Severe Anemia (Hb<8.0)	164	7.6
	Persistent Severe Anemia	14	0.7
	Malaria (slide positive)	634	29.5
	Persistent Malaria	106	4.9
	HIV Positive Status	563	26.2
	Syphilis (VDRL positive)	150	7.0
**Previous pregnancy outcome** (n = 1369, excludes primiparous women)	Previous Preterm birth	100	4.7
	Previous Stillbirth	78	3.8
	Previous Neonatal Death	65	2.9

### Factors associated with preterm compared to term births

Table 2 summarizes characteristics of women at booking who delivered at term (37–41 weeks), preterm (24–36 completed weeks), early preterm (24–33 weeks) and late preterm (34–36 weeks).

**Table 2:** Characteristics at time of booking for women who gave birth at term, preterm, early preterm and late preterm.

Study Characteristic	Characteristic subcategory	Term Birth (37–41 wks) (n = 1729)	Preterm (24–36 wks) vs. Term (n = 351)	p-value	Early Preterm (24–33 wks) vs. Term (n = 87)	p-value	Late Preterm (34–36 wks) vs. Term (n = 264)	p-value
**Age**	(mean, SD, years)	23.02 +/− 5.08	22.8 +/− 5.69	0.55	22.92 +/− 5.87	0.88	22.8 +/− 5.65	0.54
	<20 (%)	27.9	33.6	0.03	37.2	0.07	32.5	0.13
**Parity**	Primiparous (parity 0) (%)	35.8	39.8	0.18	39.7	0.48	39.8	0.23
**BMI**	(mean, SD)	22.8 +/− 2.6	22.3 +/− 2.5	0.006	22.3 +/− 2.7	0.15	22.3 +/− 2.4	0.02
	BMI <18.5 (%)	3.2	4.8	0.09	9.3	0.005	3.8	0.64
**Previous pregnancy outcome** a	Previous preterm birth (%)	6.1	13.2	0.001	19.1	0.001	11.3	0.02
	Previous stillbirth (%)	5.0	7.6	0.14	14.9	0. 003	5.3	0.87
	Previous neonatal death (%)	4.1	8.1	0.02	8.5	0.15	8.0	0.03

a Excludes primiparous women.

Women who gave birth preterm were more likely to report a history of previous preterm birth (13.2% vs. 6.1%, p = 0.001) and previous neonatal death (8.1% vs. 4.1%, p = 0.02) compared to women who delivered at term. Compared to women who gave birth at term, a significantly greater proportion of women with preterm births were less than 20 years old (33.6% vs. 27.9%, p = 0.03) and had lower mean BMI (22.3 vs. 22.8, p = 0.006).

During pregnancy (Table 3) women who had a preterm birth had lower mean weight gain (kg) between the first (booking <24 weeks gestation) and subsequent assessment (28–32 weeks gestation) (2.95 vs. 3.39, p = 0.008). More women who delivered preterm were anemic (73.5% vs. 64.2%, p = 0.001) or had malaria (36.4% vs. 28.5%, p = 0.004) at least once during their pregnancy; a significantly greater proportion also had persistent malaria (7.5% vs. 4.7%, p = 0.04). No statistical differences were noted for the prevalence of syphilis (p = 0.12) or HIV positive status (p = 0.30) between those who delivered preterm versus term.

**Table 3:** Weight gain and prevalence of morbidity during pregnancy in women who gave birth at term, preterm, early preterm and late preterm.

Study Characteristic	Characteristic subcategory	Term Birth	Preterm	P-value	Early Preterm	P-value	Late Preterm	P-value
		(37–41 wks)	(24–36 wks) vs. Term		(24–33 wks) vs. Term		(34–36 wks) vs. Term	
		(n = 1729)	(n = 351)		(n = 87)		(n = 264)	
**Weight**	Weight gain (kg: mean, SD)	3.39 (2.14)	2.95 (1.9)	0.008	3.03 (2.08)	0.37	2.94 (1.88)	0.01
**Anemia**	Ever Anemia (Hb<11.0) (%)	64.2	73.5	0.001	76.7	0.03	72.6	0.01
	Persistent Anemia	29.7	29.7	0.99	20.0	0.07	32.8	0.33
**Severe anemia**	Ever Severe Anemia	7.9	11.2	0.068	14.3	0.10	10.5	0.18
	Persistent Severe Anemia	0.5	1.2	0.18	0	0.51	1.5	0.06
**Malaria**	Ever Malaria (slide positive) (%)	28.5	36.4	0.004	32.1	0.50	37.8	0.003
	Persistent Malaria	4.7	7.5	0.04	0	0.89	8.2	0.02
**Syphilis**	VDRL positive (%)	6.5	8.9	0.12	10.3	0.19	8.4	0.26
**HIV**	Seropositive (%)	25.4	28.1	0.30	29.5	0.42	27.7	0.43

### Factors associated with early preterm compared to term births

Compared to women who delivered at term, a greater proportion of women with early preterm birth were underweight, with BMI <18.5 (9.3% vs. 3.2%, p = 0.005), and anemic during pregnancy (76.7% vs. 64.2%, p = 0.03).

More women with early preterm birth reported a previous adverse pregnancy outcome including previous preterm delivery (19.1% vs. 6.1%, p = 0.001) and previous stillbirth (14.9% vs. 5.0%, p = 0.003). Other factors that were potentially important but of borderline statistical significance (0.05<p≤0.10) included the following: adolescence (age <20) (37.2% vs. 27.9%, p = 0.07) severe anemia (14.3% vs. 7.9%, p = 0.10). There were no other notable differences.

### Factors associated with late preterm compared to term births

Compared to women who delivered at term, women who delivered late preterm had significantly lower mean BMI at booking (first antenatal visit) (22.3 vs. 22.8, p = 0.02), and gained less weight (kgs) between the booking visit and 28–32 week visit (2.94 vs. 3.39, p = 0.01). Anemia (72.6% vs. 64.5%, p = 0.01) was more prevalent among women who delivered late preterm. Similarly, malaria at any time during pregnancy (37.8% vs. 28.5%, p = 0.003) and persistent malaria (8.2% vs. 4.7%, p = 0.02) were also more prevalent among women who delivered late preterm. Late preterm birth was also associated with a history of previous preterm delivery (11.3% vs. 6.1%, p = 0.02), and neonatal death (8.0% vs. 4.1%, p = 0.03).

Association with age group approached statistical significance; age over 40 years was associated with increase in late preterm compared to term birth (2.0% vs. 0.8%, p = 0.06). Women over 40 were also more likely to report previous preterm birth (6.4% vs 4.1% p = 0.08) or a neonatal death (4.5% vs 2.6%, p = 0.08).

In this population, the prevalence of HIV was 26.2% overall (Table 1) and was not statistically different between women who delivered at term versus those who delivered preterm (early or late). While additional analyses revealed statistically significant associations between HIV and anemia and borderline statistically significant relationships between HIV and malaria, no significant interactions were found between HIV, malaria or anemia and any of the preterm birth categories.

### Multivariate analyses

Table 4 shows results of multivariate logistic regression of factors independently associated with (all) preterm birth, early and late preterm birth.

**Table 4:** Univariate and multivariate analyses for factors associated with all preterm, early and late preterm birth.

	Preterm Birth (24–36 weeks)				Early Preterm Birth (24–33 weeks)		Late Preterm Birth (34–36 weeks)	
Study Characteristic	Univariate OR, 95% CI	p-value	Adjusted OR (95% CI)	p-value	Adjusted OR, (95% CI)	p-value	Adjusted OR, (95% CI)	p-value
Age <20	1.31 (1.02–1.69)	0.03	NS		1.73 (1.03–2.90)	0.04	NS	
BMI	0.93 (0.89–0.98)	0.006	0.91 (0.85–0.97)	0.005	NS		0.91 (0.85–0.98)	0.01
BMI <18.5	1.63 (0.92–2.89)	0.09	NS		NS		NS	
Weight Gain	0.90 (0.83–0.97)	0.008	0.89 (0.82–0.97)	0.006	NS		0.89 (0.80–0.96)	0.005
Ever Anemia (Hb<11.0)	1.44 (1.12–1.84)	0.001	NS		1.95 (1.08–3.52)	0.03	NS	
Persistent Anemia (Hb<11.0 at booking visit and at 28–32 weeks )	1.36 (1.08–1.73)	0.99	NS		NS		NS	
Ever Malaria	1.44 (1.12–1.84)	0.004	NS		NS		NS	
Persistent Malaria (peripheral slide positive at booking and at 28–32 weeks)	1.75 (1.10–2.79)	0.04	1.94 (1.06–3.57)	0.03	NS		1.99 (1.05–3.79)	0.04
Previous Preterm birth	2.02 (1.27–3.22)	0.001	2.13 (1.19–3.80)	0.01	2.68 (1.10–6.52)	0.03	2.07 (1.11 – 3.86)	0.02
Previous Neonatal Death or Stillbirth	1.61 (1.05–2.49)	0.02	NS		NS		NS	

For all preterm birth, women with a history of a previous preterm delivery have more than twice the odds of having a repeat preterm birth (OR 2.13, 95% CI 1.19 – 3.80, p = 0.01).

With increasing BMI the odds of preterm birth is significantly reduced (OR 0.91, 95%, CI 0.85–0.97, p = 0.005) and this association is also seen with weight gain (OR 0.89, 95% CI 0.82–0.97, p = 0.006).

Variables independently associated with early preterm birth were; age less than 20 years, anemia and a previous preterm birth. Adolescent age (age<20) (Adjusted OR 1.73, 95% CI 1.03–2.90, p = 0.04) increased the odds of an early preterm delivery by more than 70%. Being anemic at any point during the pregnancy nearly doubled the odds of early preterm labour (Adjusted OR 1.95, 95% CI 1.08–3.52, p = 0.03). Having had a previous preterm delivery increased the odds of an early preterm delivery by more than two and a half times (Adjusted OR 2.68, 95% CI 1.10 – 6.52, p = 0.03).

Factors that remained independently associated with late preterm birth were different to those for early preterm birth and included: BMI, weight gain and previous preterm birth. We found that an increased BMI (Adjusted OR 0.91, 95% CI 0.85–0.98, p = 0.01) and weight gain (Adjusted OR 0.89, 95% CI 0.80–0.96, p = 0.005) reduced the odds of late preterm birth. Similar to early preterm, a history of previous preterm birth (Adjusted OR 2.07, 95% CI 1.11–3.86, p = 0.02) doubled the odds of late preterm birth.

## Discussion

This study reports on the factors associated with preterm birth in an unselected rural pregnant population in Malawi, a country with the highest reported rate of preterm birth worldwide and with one in four women HIV positive. To the best of our knowledge, this is the first study from sub-Saharan Africa to report on the factors associated with preterm birth for a cohort of women in which gestational age has been reliably assessed with ultrasound.

Although the incidence of preterm birth can be, as we have shown, very high in sub-Saharan Africa, there is very little data based on accurate gestational age assessment using prenatal ultrasound dating. The absence of relatively expensive ultrasound equipment is unsurprising in routine clinical assessment in low resource settings but ultrasound should be essential in future research studies of preterm birth [14]. Overall, 16.3% of women (singleton pregnancies only) included in this secondary analysis had a preterm birth with the majority of these (75%) being late preterm births between 34 and 36 weeks.

The incidence of preterm birth in our population is almost identical to recently reported, ultrasound-dated figures from a clinical trial in Botswana – 16.7% [15]. These incidences are substantially higher than figures from elsewhere in the world and deserve exploration of cause.

It has been assumed that infective morbidity (including infection with HIV) is largely responsible for higher rates of preterm birth in Africa compared with other regions [11]. In fact, we were unable to demonstrate any impact of HIV infection on preterm birth. Our study was performed at a time when there was considerable stigma associated with HIV infection in the study site community [16] and anti-retroviral (ARV) drugs were largely inaccessible in the country. Although women recruited into the study had the option of getting HIV testing and counseling, none did and we are unaware of any woman in the study taking ARV therapy during pregnancy. In accordance with the directions of the research ethics committee, we did not test blood samples for HIV status during the study. These were only tested retrospectively well after completion of the trial.

This is, therefore, a unique cohort of pregnant women with a high incidence of HIV positivity (26.2%), accurate ultrasound dating of gestational age, but no ARV treatment. In this cohort, we found no evidence that HIV status affects the risk of preterm birth. Such a study would not now be possible with the (very welcome) changes in this community of women having access to ARV treatment and therefore requesting HIV testing. Whilst there remains controversy as to whether ARVs (or certain classes of ARVs) increase the risk of preterm birth or not [17], this is a confounder that would make it impossible now to undertake a similar study to assess the direct effects of HIV infection on gestation at birth. Our finding fits with the findings of a pre-ARV study of pregnancy outcome in South Africa in which maternal HIV infection also did not increase the risk of preterm birth (although in the South African study, gestational age was not ultrasound-determined) [18].

The implication is that, whatever other advantages stem from ARV use in HIV infected pregnant women in Malawi, there is no evidence from the study suggesting that reducing the risk of preterm birth is one.

Some factors that we did find to be associated with preterm birth have been recognized in other populations. Thus, a history of previous preterm birth independently and significantly increased the odds of preterm birth overall (OR 2.13, 95% CI 1.19–3.80, p = 0.01); late preterm birth (OR 2.07, 95% CI 1.11–3.86, p = 0.02) and early preterm birth (OR 2.68, 95% CI 1.10–6.52, p = 0.03).

Similarly, persistent malaria was associated with a doubling of the risk of preterm birth.

Although up to 30% of women had peripheral malaria parasitaemia at the time of booking, all women received presumptive treatment for malaria and persistent malaria was not common in this population (5%). However, if present, persistent parasitaemia was associated with increased odds for preterm birth. There has been discussion about the adequacy of sulphadoxine-pyrimethamine intermittent preventative treatment, given increasing parasitic resistance [19] as well as whether prophylaxis should commence earlier in pregnancy, and the importance of simultaneous bed net use.

There was also an association with poor maternal nutritional state (low BMI and reduced weight gain) and, for early preterm birth, maternal anemia. We found that maternal weight played a significant role in the risk for all preterm birth, though differently for early versus late preterm. The odds of preterm birth were increased nearly three-fold for those who were underweight (BMI <18.5) at booking (even when controlled for HIV positive status), while the odds of late preterm were decreased if the patient gained weight (in between booking and the second visit) or increased her BMI, demonstrating a protective effect of weight against late preterm birth.

Results obtained in our study are similar to those reported in a recent large systematic review and meta-analysis on maternal underweight that pooled data from 52 cohort studies and 26 case control studies mostly from developed countries and showed an increased risk of preterm birth in underweight women [20]. An increased risk of preterm birth in association with low BMI has been described in the UK (and elsewhere) as an independent factor alongside social deprivation and smoking [21].

These findings raise the question of whether preterm birth can be prevented by improving maternal nutrition. A Cochrane review [22] identified 5 trials, involving 3384 women, of nutritional supplementation with preterm birth as an outcome measure; the effect (relative risk 0.96, 95% CI 0.80, 1.16) did not suggest benefit but only two of the trials took place in low income countries and only one of these was in Africa [23]. The possibility of benefit from better nutrition therefore remains an open question, suitable for future research.

The mechanisms are unclear but both low BMI and anemia may have common cause in poor nutrition or chronic infection or both. Maternal anemia is recognized as an important risk factor for the mother, particularly if she has a postpartum haemorrhage. Our findings suggest that maternal anemia should also be recognized as a risk factor for preterm birth.

All women who took part in this study attended for antenatal care on at least one occasion but the study did not include women who did not access care until after 24 weeks or who did not access antenatal care at all. However, in this setting, more than 90% of pregnant women do attend for antenatal care [24] and we believe this cohort is representative of the population in many similar settings in sub-Saharan Africa.

Because HIV testing was performed retrospectively on stored samples, CD4 counts were not obtained and no information was available about severity of HIV infection. Parasitic infection (other than malaria) was not assessed in this cohort. We have previously noted that hookworm and other parasites were uncommon in this population [25]. Similarly, we were unable to test for urinary tract infections or sexually transmitted infections other than HIV and syphilis in this cohort at the time. Further research is needed to assess the burden of co-morbidities in pregnant women in this type of setting with an examination of the relationship of these with pregnancy outcome.

## Conclusions

Preterm birth remains a significant risk factor for neonatal mortality. Developing a deeper understanding of the factors significantly associated with preterm birth in a community with an extremely high incidence and particularly identifying those factors that are modifiable, could help develop new approaches to antenatal (and pre pregnancy) care to prevent adverse pregnancy outcome. Our findings have underscored the importance of women's pregnancy history and identified maternal underweight, malaria and anemia as risk factors for preterm birth. Unexpectedly, we found no evidence that HIV status contributes to the risk of preterm birth.
